# Applicability of the MASK-Air^®^ App to Severe Asthma Treated with Biologic Molecules: A Pilot Study

**DOI:** 10.3390/ijms231911470

**Published:** 2022-09-29

**Authors:** Alida Benfante, Bernardo Sousa-Pinto, Gianluca Pillitteri, Salvatore Battaglia, Joao Fonseca, Jean Bousquet, Nicola Scichilone

**Affiliations:** 1 PROMISE—Dipartimento di Promozione della Salute, Materno-Infantile, di Medicina Interna e Specialistica di Eccellenza “G. D’Alessandro”, University of Palermo, Piazza delle Cliniche n. 2, 90127 Palermo, Italy; 2 MEDCIDS—Department of Community Medicine, Information and Health Decision Sciences, Faculty of Medicine, University of Porto, 4200-450 Porto, Portugal; 3 CINTESIS—Center for Health Technology and Services Research, University of Porto, 4200-450 Porto, Portugal; 4 RISE—Health Research Network, University of Porto, 4200-450 Porto, Portugal; 5 MACVIA-France, 34000 Montpellier, France; 6 Department of Dermatology and Allergy, Comprehensive Allergy Center, Charité, Universitätsmedizin Berlin, Humboldt-Universität zu Berlin, 10099 Berlin, Germany; 7 Centre Hospitalier Universitaire, 34295 Montpellier, France

**Keywords:** telemedicine, application, visual analogue scales, severe asthma, biologic molecules

## Abstract

MASK-air^®^, a good practice of the DG Santé, has been fully validated in allergic rhinitis, but little is known about its applicability to asthmatics. We explored whether the MASK-air^®^ application is applicable to patients with severe asthma. Severe asthmatics were proposed to use the MASK-air^®^ application for 6 months, along with best practice treatment. Treatment of the patients was not changed based on the application results. The evolution of the visual analogue scales (VAS) for asthma, shortness of breath, rhinitis, conjunctivitis, work, and sleep was monitored using MASK-air^®^. Adherence to MASK-air^®^ and to the asthma treatment was also checked. Thirteen patients reported on 1229 days of MASK-air^®^ use. The average application adherence was 51.8% (range: 19.7–98.9%). There was no correlation between application and medication adherence. Highly variably trends were found for the VAS for asthma. Five patients had over 90% well-controlled days, four had well- or moderately controlled asthma (with up to 20% uncontrolled days), one patient had moderately controlled asthma with approximately 20% uncontrolled days, and one patient had 80% uncontrolled days. Highly significant correlations were found for the VAS for asthma, and other patients reported VASs for work, dyspnea, sleep, and rhinitis. MASK-air^®^ can be used in patients with severe asthma. VAS asthma appears to be an interesting patient-reported outcome highly correlated with dyspnea and impacts on work. Adherence to the application was better than that for rhinitis, but it needs to be improved.

## 1. Introduction

The goal of asthma management is to reach the optimal control of respiratory symptoms, limiting their impacts on daily activity and quality of life [1]. However, these goals are not always achieved in patients with severe asthma. Recently, novel strategies and approaches have been adopted to treat and monitor severe asthmatic patients who are regularly followed at dedicated outpatient clinics. These include the use of telemedicine, which has proven to be particularly useful in the management of patients with chronic diseases (such as asthma) who need continuous monitoring.

A study comparing telemedicine with face-to-face visits showed equal levels of disease control, suggesting that telemedicine may be considered a valid alternative [2]. Another of these novel approaches is mobile health (mHealth), which includes applications running on consumer smart devices (i.e., smartphones and tablets), and it is becoming increasingly popular [3]. These application-based collaborative systems allow for gathering clinical information quickly, thus permitting early initiatives by the patients and proper interventions by the physicians. While mHealth applications appear effective for the self-management of asthma [4,5], further results are needed [6,7] as some studies have been completed in a randomised fashion and not with real world data [8,9].

In 2014, on behalf of the European Innovation Partnership on Active and Healthy Ageing (EIP on AHA) [10], the AIRWAYS ICP (Integrated Care Pathways for airway diseases, the DG Santé and DG CNECT) was initiated [11]. The objective was to develop digitally enabled multi-sectoral care pathways (ICPs) for chronic respiratory diseases. MASK (Mobile Airways Sentinel NetworK) is the mHealth strategy of AIRWAYS ICPs and ARIA [12]. MASK-air^®^ has been developed, in collaboration with professional and patient organizations, as an application centered around the patient, and it is currently operational in 27 countries, using 20 languages, with over 40,000 users, and is a good practice for the digitally enabled, patient-centered care of the DG Santé in rhinitis and asthma multimorbidity [13]. A transfer of innovative practices (TWINNING) was performed to transfer and implement MASK-air^®^ to 22 countries or regions [14].

Most results published using MASK-air^®^ data concern in rhinitis. In fact, although MASK-air^®^ displays a large amount of data on patients with asthma, little is known about the applicability of the MASK-air^®^ application to asthmatic patients [13]. A recent study by Ventura et al. found that older asthmatic adults with a low level of education are able to effectively use the MASK-air^®^ application after a short training session [15], improving the management and treatment of their geriatric asthma [16,17]. In addition, a study by Sousa-Pinto et al. aimed to assess the correlation between VAS asthma and other MASK-air^®^ daily reported PROMs (patient-reported outcome measures) in asthmatic patients with nasal symptoms requiring the Global Initiative for Asthma (GINA) 4 or 5 level of medications [18].

Given the potential of the MASK-air^®^ application, there is an urgent need to validate it in the subgroup of subjects affected by the most severe forms of asthma who are treated with biologic molecules. Therefore, we explored whether the MASK-air^®^ app is applicable to severe asthmatics.

## 2. Results

A total of 15 patients were consecutively enrolled, and 13 used the MASK-air^®^ application (M/F: 4/11, age range: 18–66 years) (Table 1). All of them suffered from allergic rhinitis, and 12 were using biological drugs for severe asthma. During the 6-month interval, subjects regularly attended the follow-up visits and no complaints were reported.

**Figure 1:**
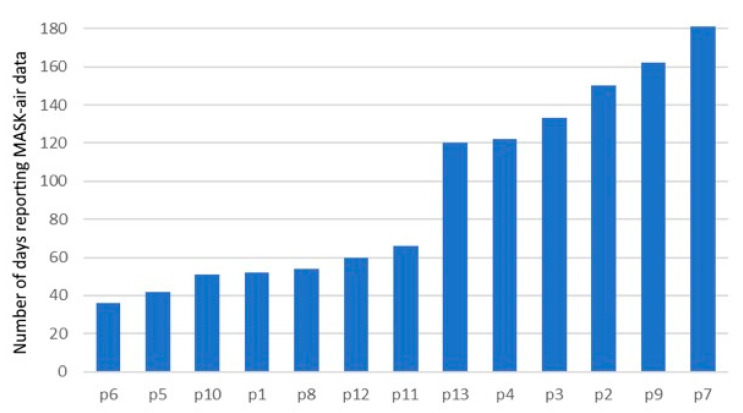
Adherence to the application.

Among the 13 patients who used the application, a total of 1229 days were reported (median, percentile 25–75: 93, 53–137 days per patient). The average application adherence was of 51.8% (range: 19.7–98.9%). Three patients had an application adherence of over 80%, three had between 60% and 80%, and all the others had under 40% (Figure 1). Most patients used the application discontinuously. In addition, in the enrolled patients, the use and adherence of the application did not seem to be affected by age, per se.

The median mMPR was 96.7%, with mMPRs ranging from 60.6% to 100%. Only one patient had an mMPR of lower than 80% (Table 2). The percentage of days for which patients reported only their usual asthma long-acting treatment (with no additional asthma medication) ranged from 48.1% to 100% (median score of 91.8%). Overall, the mMPR displayed a poor correlation with application adherence (Spearman’s rank correlation coefficient of 0.132).

**Figure 2:**
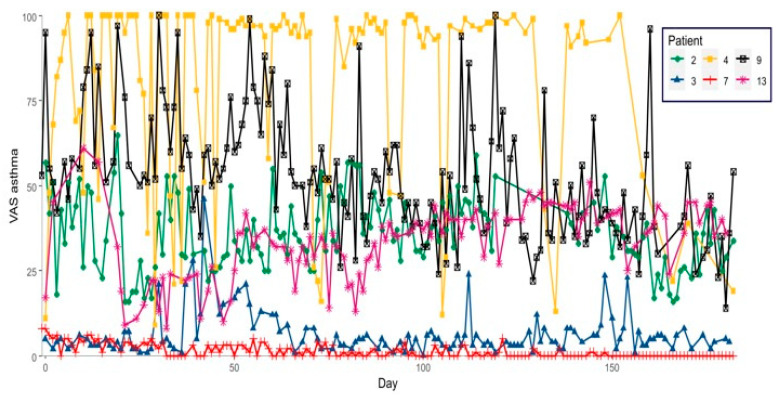
Trends of VAS asthma for patients with adherence of >50%.

**Figure 3:**
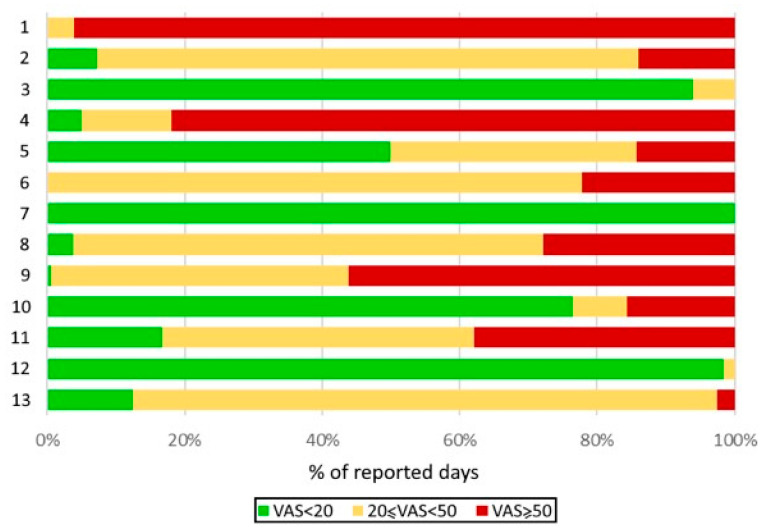
Distribution of well-controlled, moderately controlled, and poorly controlled days by patient, as assessed by the visual analogue scale (VAS) quantifying the severity of asthma symptoms.

Highly variably trends were found in these patients for VAS asthma (Figure 2). Five patients had over 90% well-controlled days and only one had no exacerbation (Figure 3). Four patients had well- or moderately controlled asthma, and all had up to 20% uncontrolled days. One patient had moderately controlled asthma with approximately 20% uncontrolled days, and one patient had 80% uncontrolled days.

**Figure 4:**
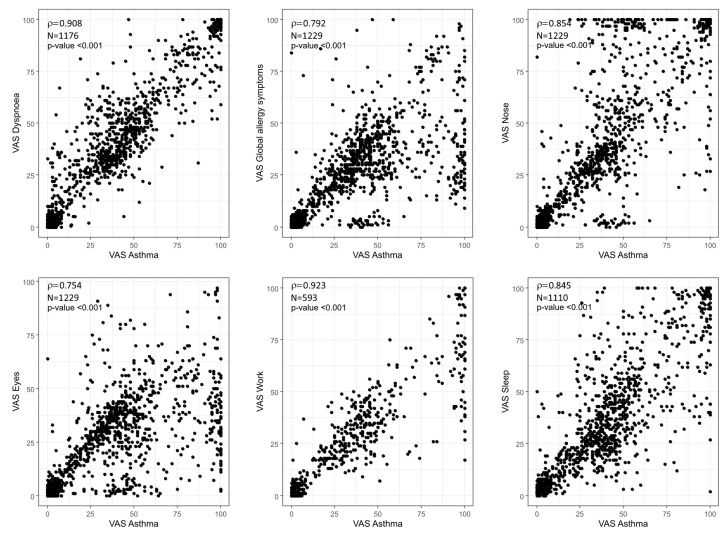
Correlations between different visual analogue scales (VAS) and the VAS assessing the severity of asthma symptoms (“VAS Asthma”) (Spearman’s rank correlation). N: number of observations; ρ = Spearman’s rank correlation coefficient.

We observed strong correlations between all VAS asthma values considered in the 1229 days reported by the patients (Figure 4) (the number of observations was identical for most correlations, except for the VAS work since there were many days when the users did not work). All VAS values had a Spearman’s rank correlation coefficient of over 0.75 when compared with the VAS asthma, with that between the VAS dyspnea and VAS asthma being 0.908.

## 3. Discussion

The current study shows that a simple patient-reported outcome (PRO) (VAS asthma) can assess the daily control of asthma. Despite advances in knowledge on the disease and wider access to novel drugs [19], a significant proportion of asthmatic patients remains inadequately controlled, thus increasing the costs for society and affecting health-care resources [20,21]. Among the potential reasons, the lack of integrated care pathways and the lack of patient empowerment should be recognised. It has been demonstrated that closer monitoring of the disease through the use of tools to check symptoms (questionnaires) and/or lung function (peak expiratory flow meters) variability or simply by educating the patient to follow recommendations and to adhere to guidelines improves the level of disease control. In this scenario, the use of mHealth and real-time data management with an innovative investigatory approach could contribute to better characterise the disease and improve adherence. A body of literature has supported the validity of the MASK application as a tool to improve the control of nasal and ocular symptoms [22] and to assess adherence to treatment in rhinitis [23]. However, little is known about the applicability of the application to asthmatic patients with concomitant rhinitis. In a recent study, this mHealth application was shown to be effective for the self-management of allergic rhinitis and/or asthma [20]. We therefore conducted a pilot study with the aim of investigating whether the MASK-air^®^ is applicable to the most severe forms of asthma.

MASK-air^®^ was used to collect daily VAS data for allergic symptoms and VAS nasal, ocular, asthma, shortness of breath symptoms and VAS work in a real-life setting. In addition, the application adherence/intensity of use and medication adherence were recorded. The present study showed that MASK-air^®^ can be efficaciously used in patients with severe asthma, thus supporting the use of daily assessment to improve outcomes and quality of care. The VAS asthma was found to be highly correlated with the VAS dyspnea and VAS work, appearing to be an interesting PRO. Adherence to the MASK-air^®^ application for these severe asthmatics was found to be better than that in allergic rhinitis, although efforts are needed to improve adherence.

This study has limitations, the most important of them being of the small sample size of the study, with only 13 patients studied. Another limitation is the possible overestimation of MASK-air^®^ adherence. In fact, the included patients were proposed to use MASK-air^®^ and were closely followed by their physicians, factors which may have resulted in increased MASK-air^®^ adherence. Other limitations include the possibility of selection bias (as (i) only patients from a single clinic were assessed, and (ii) those 13 patients who accepted using MASK-air^®^ may not have been representative of all patients with severe asthma) and incorrect information being provided about MASK-air^®^. Finally, since the recruitment phase lasted one year and the use of the application was planned for 6 months, climate changes could have affected the results of the study. The influence of climate changes should be included in the interpretation of the data.

When we evaluated trends of the VAS asthma for patients with asthma adherence of >50%, inter- and intra-subject highly variable trends were recorded, according to the well-known heterogeneity and variability of severe asthma [24]. This suggests a need for a daily assessment of control and medications. The average application adherence was 51.8% and half of the enrolled patients had an inadequate application adherence (that is, under 40%). The adherence to the application was better than that of patients with rhinitis (median: 17 days) and better than that in Lithuania, where patients with rhinitis and/or asthma enrolled by physicians reported a median adherence of 54 days. This suggests that increased adherence to an application is related to its administration by physicians but possibly also to the severity of the disease. However, there was no correlation between adherence to the application and to medications (overall, high medication adherence was recorded, with a median of 91.8%). Although mHealth applications targeting medication adherence may be useful tools for helping patients take their medications as prescribed [25,26,27], the current MASK-air^®^ features have not yet been designed to improve adherence. This will be available when MASK-air becomes a class 2A medical device.

This study has important strengths. In particular, all the assessed patients had severe asthma, as confirmed by a physician, and were assessed longitudinally regarding their reported asthma symptoms. All of them were taking biological drugs for severe asthma (step 5 of the disease, according to the Global Initiative for Asthma).

The most recent study evaluating the applicability of MASK-air^®^ for the management of asthmatic subjects included moderate to severe asthmatics [18] or asthmatic patients aged between 65 and 90 years with different degrees of disease severity [15]. Although this was a pilot study, interesting information was obtained. The current results confirm the applicability of MASK-air^®^ PROs to severe asthma. The VAS asthma was highly correlated to the VAS dyspnea, suggesting that the VAS dyspnea does not provide additional information in MASK-air^®^ even for the severe form of asthma. The VAS asthma is also highly correlated with the other PROs assessed in the study, particularly the VAS work and the VAS sleep. The study was not designed to determine whether technology-based interventions can improve asthma management by facilitating patient education, symptom monitoring, environmental trigger control, comorbid condition management, and medication adherence [28]. A future study specifically designed for these purposes should be considered as the results of previous studies were inconclusive [29]. In this set of unselected severe asthma patients, we observed that there was almost no asthma without nasal symptoms (rhinitis or rhinosinusitis) and that worsening of symptoms was often accompanied by worsening nasal and bronchial symptoms. This study is another piece of the GINA puzzle about the links between the lower airways and the nose. Future studies that include a larger sample of severe asthmatic subjects are needed to assess the properties of MASK-air^®^ patient-reported outcome measures in severe asthma.

## 4. Materials and Methods

### 4.1. Study Design

Patients with severe asthma, who under the best practice treatment, were proposed to use the MASK-air^®^ application for 6 months. The MASK-air^®^ application comprises seven mandatory symptom daily monitoring questions whose responses are provided by means of a VAS. To assess the control of asthma, the VAS cutoffs used in previous rhinitis studies (0–19: well-controlled, 20–49: moderately controlled, and ≥50: poorly controlled) [30] were used. In addition, if users reported that they were working, they were asked “how much allergic symptoms affected work activities on that day” (VAS work). Treatment was not changed based on the application’s results. Patients were then subjected to regular, continuous follow-ups, with a consultation every 2 months per the regular follow-up protocol of the clinic. The evolution of the visual analogue scales (VAS) for asthma, shortness of breath, rhinitis, conjunctivitis, work, and sleep, was monitored.

### 4.2. Setting

The study took place at the severe asthma clinic of the University Hospital of Palermo, Italy.

### 4.3. Participants

In this pilot study, we aimed to assess the usability of the MASK-air^®^ outcomes in severe asthma to a limited number of patients. The results of the study will be used to assess the number of subjects required for further studies. Consecutive asthmatic patients regularly attending a single outpatient clinic from June 2019 to June 2020 and who were diagnosed with the most severe form (step 5) of the disease, according to the Global Initiative for Asthma (GINA) [1], and were using biological drugs for severe asthma were invited to participate to the current pilot experiment. At the first of their regular follow-up visits, the subjects who agreed to participate and gave their consent were trained to freely download and use the application. Each subject was instructed to access the application and complete all the fields daily for a period of 6 months. The terms of reference followed the GDPR and the country’s legislation [31], and they allowed the use of the results for research purposes. The data are anonymised, including all geolocalised data. An Independent Review Board approval was not needed as this was an observational study without any change in the patients’ management.

There is no pulmonary function test in the current MASK-air^®^ application since asthma control scores do not usually include such information, and the addition of FEV_1_ values does not improve the effectiveness of the control test [32].

### 4.4. Data Analysis

The categorical variables were described using absolutes and relative frequencies. For the continuous variables, since MASK-air^®^ VASs do not follow a normal distribution, medians and percentiles were used. The Spearman’s rank correlation test was used for correlating (i) different VASs with each other, and (ii) application adherence with medication adherence. Application adherence/intensity of use was defined following the methods of Di Fraia et al., being calculated as the number of actual reporting days divided by the days in the reporting period (182 or 183 days, depending on the time period, corresponding to a period of 6 months) [33]. Medication adherence was assessed according to the modified medication possession ratio (mMPR), as defined by Menditto et al. [23], namely, by dividing the number of days of reported asthma long-acting treatment by the number of MASK-air^®^ reporting days. For this estimation, (i) for each day, medication adherence was assumed to be observed if the patient reported all asthma long-acting drugs in his/her treatment plan (such plan, however, was allowed to vary without patient exclusion), (ii) biologics were not taken into account, as they are not used on a daily basis, and (iii) variation in the treatment for rhinitis was not considered.

## 5. Conclusions

We demonstrated that the MASK-air^®^ application is applicable to asthmatics with the most severe forms of disease. The current information sets the basis for larger investigations of the MASK-air^®^ application to this population to assess whether it may contribute to improvements in asthma control and adherence to treatment.

## Figures and Tables

**Table 1:** Demographic characteristics of the patients.

Patient	1	2	3	4	5	6	7	8	9	10	11	12	13
Age	44	43	37	21	55	56	59	66	41	18	48	59	62
Sex	M	M	F	F	M	F	F	M	F	F	F	F	F
GINA step	5	5	5	5	5	5	5	5	5	5	5	5	5
Rhinitis	+	+	+	+	+	+	+	+	+	+	+	+	+
CRSwNP		+			+EGPA		+			+	+	+	
Conjunctivitis		+											+
OSAS	+	+											
FEV_1_ (% pred)	80	101	83	91	71	89	40	122	125	82	73	70	104
FEV_1_/FVC	55	125	84	112	62	75	50	107	106	103	90	105	99
Eos/mm^3^	100	700	300	60	0	170	30		950	160	0	760	250
Total IgE	25	761	300	84	15	27	238	30	20	518	130	66	81
ICS/LABA	+	+	+	+	+	+	+	+	+	+	+	+	+
LAMA	+		+		+	+	+				+		
Other meds		+	+		+			+	+	+			
Omalizumab			+	+						+			
Mepolizumab	+	+				+		+					
Benralizumab					+		+		+		+	+	
Dupilumab													+

**Table 2:** Frequency of medication use.

Patient Number	Number of MASK-Air^®^ Reporting Days	Number of Days Reporting Treatment with the Usual Asthma Long-Acting Medication (% ^a^)	Number of Days Reporting Treatment with the Usual Asthma Long-Acting Medication, with No Additional Asthma Medication Used (%)
6	36	32 (88.9)	32 (88.9)
5	42	40 (95.2)	40 (95.2)
10	51	51 (100)	37 (72.6)
1	52	51 (98.1)	25 (48.1)
8	54	54 (100)	54 (100)
12	60	58 (96.7)	58 (96.7)
11	66	40 (60.6)	40 (60.6)
13	120	96 (80.0)	93 (77.5)
4	122	117 (95.9)	112 (91.8)
3	133	127 (95.5)	126 (94.7)
2	150	149 (99.3)	140 (93.3)
9	162	159 (98.2)	133 (82.1)
7	181	177 (97.8)	177 (97.8)

^a^ Corresponds to the modified medication possession ratio.

## Data Availability

All data generated or analysed during this study are included in this published article.

## Abbreviations

AHA	Active and Healthy Ageing
CRSwNP	Chronic rhinosinusitis with nasal polyps
EGPA	Eosinophilic granulomatosis with polyangiitis
EIP	European Innovation Partnership
FEV_1_	Forced expiratory volume in the first second
FVC	Forced vital capacity
GINA	Global Initiative for Asthma
ICPs	Integrated Care Pathways
ICS	Inhaled corticosteroids
LABA	Long-acting beta-agonist
LAMA	Long-acting muscarinic agonist
MASK	Mobile Airways Sentinel NetworK
mMPR	modified medication possession ratio
OSAS	Obstructive Sleep Apnea Syndrome
PRO	patient-reported outcome
SIgE	Serum specific IgE
VAS	visual analogue scales
